# Medical College Notes

**Published:** 1889-09

**Authors:** 


### Medical College Notes.

The College of Pharmacy.—The announcement of the Buffalo College of Pharmacy has been received, and several interesting features are noticed. The three senior members of the original faculty have either retired or been called to a larger field of labor, but they remain identified with the college as follows: Professor Witthaus, of New York, as Emeritus Professor of Pharmaceutical Chemistry and Toxicology; Professor D. S. Kellicott, of Columbus, O., as Emeritus Professor of Botany and Microscopy, and Dr. E. V. Stoddard, of Rochester, as Emeritus Professor of Materia Medica.

Professor Vandenbergh, who formerly had the chair of General and Analytical Chemistry, has succeeded Professor Witthaus; Dr. Ernest Wende, of this city, has been promoted from Instructor to Professor of Botany and Microscopy. The instruction in Professor Stoddard's department is to be given by a pharmacist and a physician jointly, Dr. John R. Gray giving instruction in Pharmacognosy, and Dr. Eli H. Long being Lecturer on Materia Medica.

Professor Oscar Oldberg, of Chicago, is to give a special course of suggestive lectures on the Seventh Revision of the Pharmacopeia. Dr. Vandenbergh has been elected Dean of the Faculty, Dr. Wende Registrar, and Dr. Gregory continues as Secretary and Treasurer. A laboratory of pharmacognosy has been provided for, and the instruction in every department has been increased as much as practicable.

The Junior course shows an increase from 253 hours to 275 hours actual instruction, and the senior course from the 242 hours last year to 275 hours this year. Fully half of the instruction is now given in the laboratories.

